# Gene expression profiling suggests differences in molecular mechanisms of fin elongation between cichlid species

**DOI:** 10.1038/s41598-019-45599-w

**Published:** 2019-06-21

**Authors:** Ehsan Pashay Ahi, Florian Richter, Laurène Alicia Lecaudey, Kristina M. Sefc

**Affiliations:** 1 Institute of Biology, University of Graz, Universitätsplatz 2, A-8010 Graz, Austria; 2 Department of Comparative Physiology, Uppsala University, Norbyvägen 18A, SE-75 236 Uppsala, Sweden

**Keywords:** Morphogenesis, Self-renewal, Evolutionary genetics, Molecular evolution

## Abstract

Comparative analyses of gene regulation inform about the molecular basis of phenotypic trait evolution. Here, we address a fin shape phenotype that evolved multiple times independently across teleost fish, including several species within the family Cichlidae. In a previous study, we proposed a gene regulatory network (GRN) involved in the formation and regeneration of conspicuous filamentous elongations adorning the unpaired fins of the *Neolamprologus brichardi*. Here, we tested the members of this network in the blockhead cichlid, *Steatocranus casuarius*, which displays conspicuously elongated dorsal and moderately elongated anal fins. Our study provided evidence for differences in the anatomy of fin elongation and suggested gene regulatory divergence between the two cichlid species. Only a subset of the 20 genes tested in *S. casuarius* showed the qPCR expression patterns predicted from the GRN identified in *N. brichardi*, and several of the gene-by-gene expression correlations differed between the two cichlid species. In comparison to *N. brichardi*, gene expression patterns in *S. casuarius* were in better (but not full) agreement with gene regulatory interactions inferred in zebrafish. Within *S. casuarius*, the dorsoventral asymmetry in ornament expression was accompanied by differences in gene expression patterns, including potential regulatory differentiation, between the anal and dorsal fin.

## Introduction

Gene regulatory networks (GRNs) describe the regulatory relationships among genes involved in the development and maintenance of phenotypes. A large body of work concentrated on selected developmental models, for instance sea urchins^1^, and synergistically constructed extensive GRNs including hundreds of transcription factors and signaling proteins. Additionally, comparative studies of gene regulation across different levels of biological organization and diversification (organs, individuals, populations, species) address the divergence and conservation of regulatory networks and their roles in evolutionary processes^2–4^. For instance, parallel phenotypic variation across populations or species may be controlled by parallel regulatory changes, as demonstrated in the adaptive evolution of stickleback populations^5,6^ and for leaf shape complexity in *Solanum*^7^. In contrast, distinct changes in gene expression patterns were shown to underlie similar pigmentation patterns across *Drosophila* species^8^.

**Figure 1:**
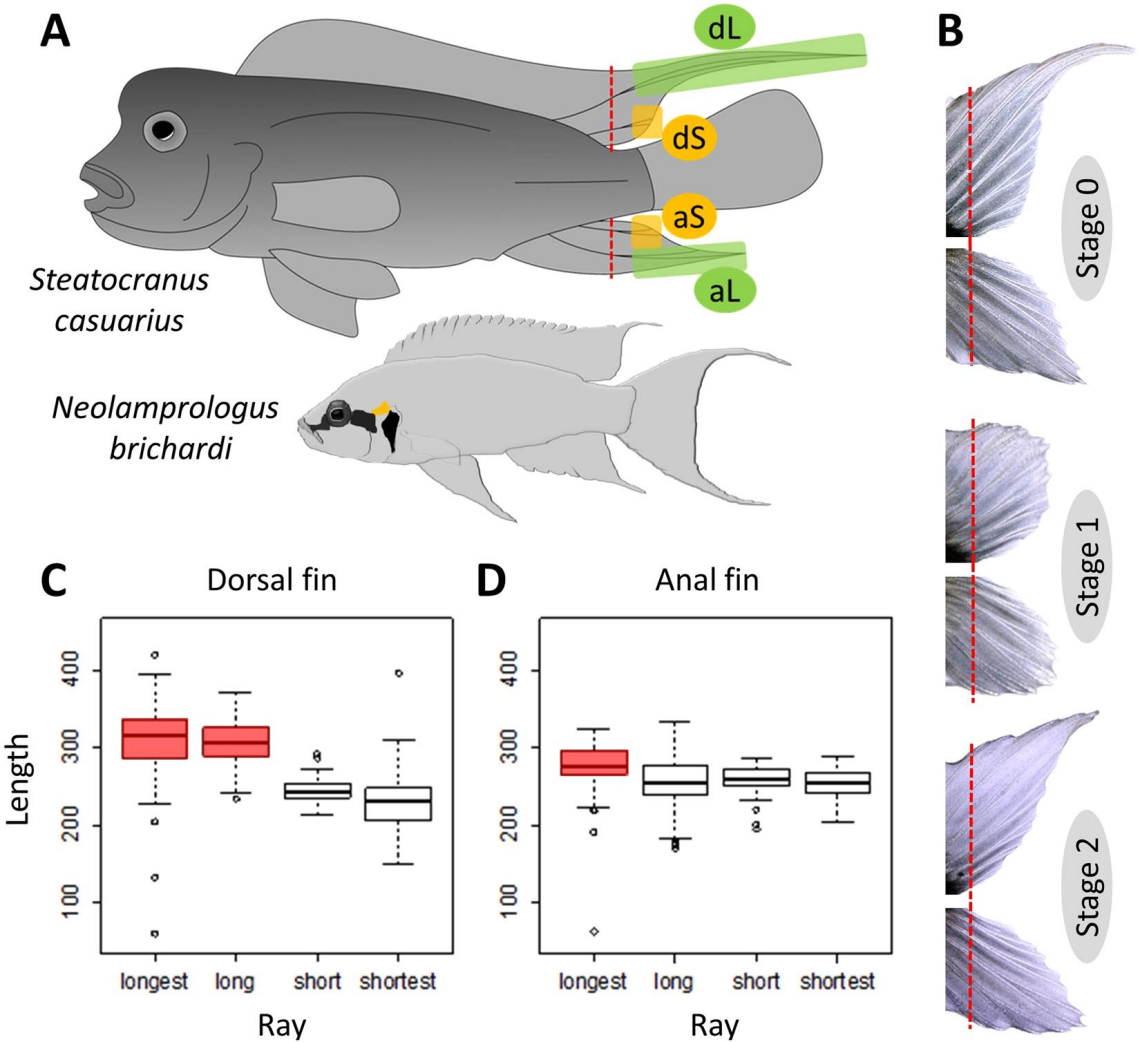
Fin elongation phenotype and sampling. (**A**) Adult male *S. casuarius* (top) grow long filamentous elongations on their dorsal and more moderate elongations on their anal fins. In contrast, fin elongations in *N. brichardi* (bottom) are similarly developed in all unpaired fins. The two species were not drawn to scale. Fins of *S. casuarius* were amputated along the dashed red line. Green shaded areas indicate tissue representing the elongated regions of the dorsal fin (dL) and anal fin (aL); yellow shaded areas indicate tissue representing the short regions of the dorsal fin (dS) and anal fin (aS). Note that the actual tissue samples comprised two fin rays each. (**B**) Biopsies were taken from the original (stage 0) and regenerating fin tissue (stage 1, 20 days of regeneration and stage 2, 40 days of regeneration) from the dorsal (top fin) and anal fin (bottom fin of each pair). The photograph representing stage 0 shows the smallest individual with the least elongated fins among the fish included in the experiment (**C,D**) Fin ray segment length in elongated (long rays) and short regions of dorsal and anal fins. Within fin type, different box colours indicate significant differences in segment length.

In fish, the molecular mechanisms controlling fin growth and regeneration have received particular attention not least due to their relevance for biomedical research^9^. Most studies focussed on the caudal fin of the zebrafish *Danio rerio*, which exhibits a particularly well developed regeneration capacity^9^. An additional reason to study the caudal fin of fishes in a comparative approach is to gain understanding about the evolution of body shape, for instance regarding the transition from the dorsoventrally asymmetrical (heterocercal) caudal fin of acipenseriformes, gars and bowfin to the outer symmetry displayed by the homocercal caudal fins of teleost fish^10^. Furthermore, within teleosts, variation in the outer shape and anatomy of caudal fins provided opportunities to identify genes and molecular mechanisms involved in fin shape formation^11–13^. The examination of dorsoventral (a)symmetry in fish fin morphology can be extended to the dorsal and anal fins, e.g. by comparing the molecular correlates of ornamental filaments^14^ or colour patterns^15^ between these fins. In a previous study, we investigated the molecular mechanisms involved with the formation of elongated fin filaments, which adorn the dorsal, caudal and anal fins of the cichlid fish *Neolamprologus brichardi*^14,16^. The extent of the outgrowth is similar across the unpaired fins in this species (Fig. 1A). Using qPCR assays of candidate genes associated with ontogenetic and regenerative fin growth in the zebrafish^14^ as well as analyses of co-expressed genes and predicted transcription factors^16^, we identified potential members of a gene regulatory network involved in the formation of the ornamental fin phenotype. The GRN had a core module of tightly coexpressed genes containing some functionally characterized members such as *cx43*, *sema3d* and *mmp9*, which have important roles in the formation and growth of fin ray segments in zebrafish^17–20^. The coexpression module further included genes (e.g. *angptl5*, *angptl7*, *dpysl5a*, *csrp1a* and *cd63*), which are required for angiogenesis or neurogenesis during fin formation^11,21–24^. Moreover, we identified six potential upstream regulators of the coexpression module (*egr2*, *foxc1*, *foxd3*, *foxp1*, *irf8* and *myc*)^16^, among which *myc*, *irf8* and *foxd3* were already indicated in fin/tissue regeneration studies^11,25–28^. Importantly, the expression patterns of *foxd3* and its significant expression correlation with all members of the coexpression module led us to conclude that *foxd3* is a main upstream regulator of the GRN in *N. brichardi*^16^. The expression patterns of some of the network genes in *N. brichardi* differed from expectations based on the functions of these genes in zebrafish. These include *cx43*, which, in zebrafish, is related with fin ray segment growth and the regulation of *mmp9* and *sema3d*^17–20^. In *N. brichardi*, differences of *cx43* expression levels between fin regions were not associated with differences in segment length, and correlations with *mmp9* and *sema3d* were in the opposite directions than in zebrafish^14,16^.

In the present study, we tested the members of the GRN in another cichlid fish, the blockhead cichlid *Steatocranus casuarius*. This species is particularly interesting because the unpaired fins of males differ with respect to the presence and extent of filament elongation (Fig. 1A). The dorsal fin displays a conspicuous elongation comparable to that seen in *N. brichardi*, whereas the extension of the anal fin is much more moderate. The caudal fin is rounded and develops no elongation at all. *S. casuarius* and *N. brichardi* are separated by approximately 15 mio years of divergence and evolved fin elongations independently^29^. We used the same experimental design as in our work with *N. brichardi* and compared the expression levels of 20 GRN genes between the elongated (L) and the short (S) regions of the anal and the dorsal fins, using both intact and regenerating fins. All of these genes had shown significant L/S expression differences in *N. brichardi*, which suggested that their differential expression was associated with the growth pattern producing the fin elongations. Furthermore, to investigate the regulatory linkages inferred in *N. brichardi*, we tested for pairwise gene expression correlations in the anal and dorsal fins of *S. casuarius*. We predicted that the L/S expression differences and the gene expression correlations observed in *N. brichardi* would be detected in the distinctly elongated dorsal fin of *S. casuarius*, but not necessarily in its anal fin. We complemented the genetic analyses of fin elongation with an anatomical characterization of the phenotype, and measured fin ray segment length in elongated and short fin regions to determine whether elongation of rays was associated with lengthening of ray segments. Our findings suggest divergence in the regulation of the fin elongation phenotype between the two cichlid species.

## Results

### Characterization of fin morphology

We measured the lengths of fin ray segments in elongated and short regions of the anal and dorsal fins in order to determine whether fin elongation was associated with an elongation of ray segments. Fin ray segments differed in length both between and within fins. The segments of the long rays were longer in the dorsal fin than in the anal fin (longest rays of each fin: t = 3.54, p = 5.59 * 10^−4^, second-longest rays: t = 8.57, p = 1.32 * 10^−13^; Fig. 1C,D). In contrast, the segments of the two short rays were shorter in the dorsal fin than in the anal fin (2^nd^-shortest rays of each fin: t = −4.15, p = 6.36 * 10^−5^, 3^rd^-shortest ray: t = −3.86, p = 1.85 * 10^−4^; Fig. 1C,D). Within the dorsal fin, the two long rays had longer segments than the two short rays, whereas in the anal fin, only the segments of the very longest ray were significantly longer than those of the remaining rays (Fig. 1C,D; Supplementary Table 1).

### Reference gene validation

**Figure 2:**
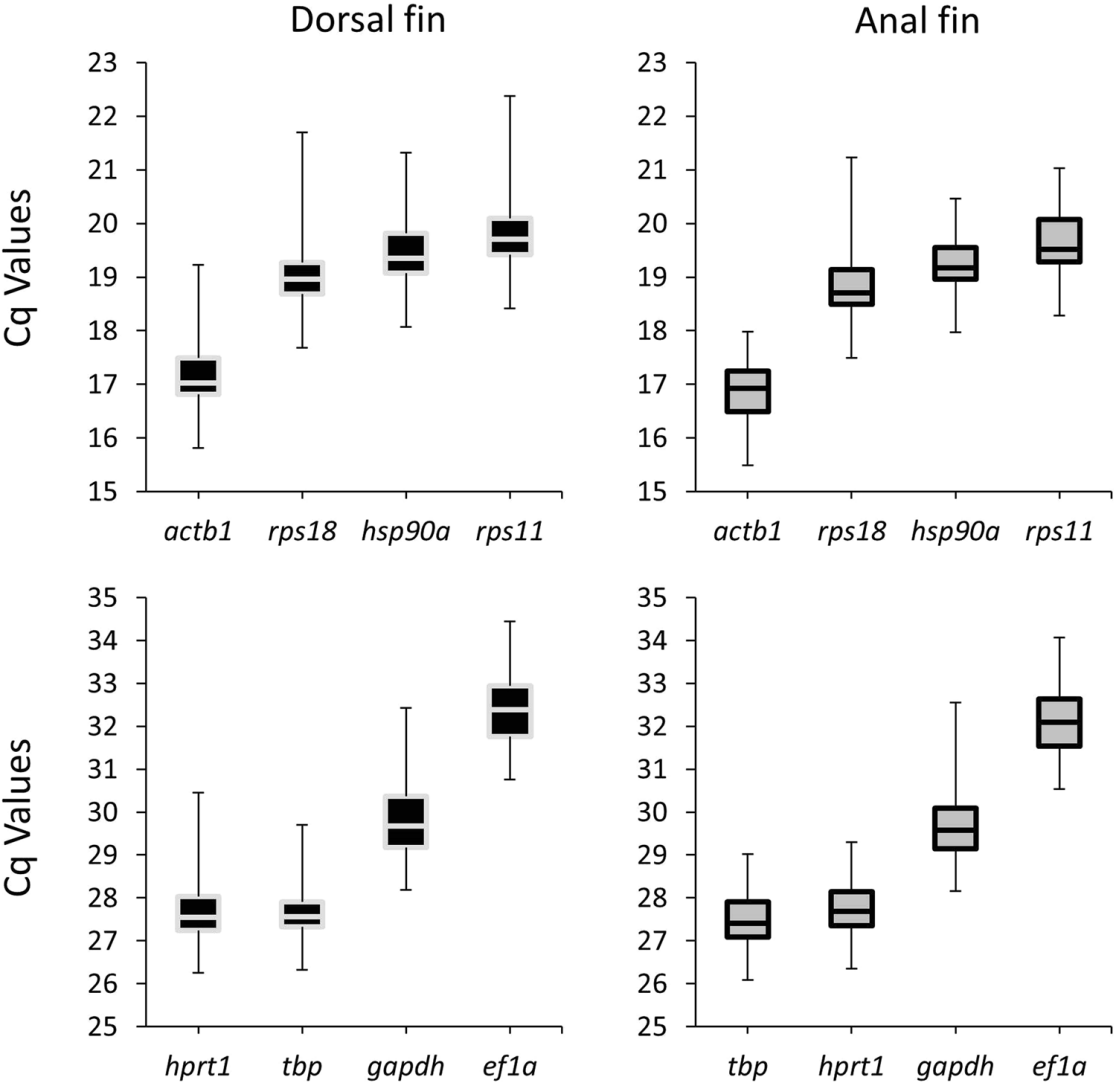
Expression levels of candidate reference genes. The black-filled (left) and grey-filled (right) boxes denote expressions in dorsal and anal fins, respectively.

The identification of reference genes with stable expression under the specific experimental conditions a precondition for the analysis of relative gene expression levels^30–32^. We tested the expression of 8 candidate reference genes in the dorsal and anal fin samples at the three regeneration stages. Congruent profiles were observed in the two fins, with *actb1* showing highest expression (lowest Cq) and *elf1a* with lowest expression (highest Cq) (Fig. 2). In the anal fin, all three algorithms ranked *hsp90a* as the most stable reference gene followed by *actb1* as the second-best validated gene (Table 1). In contrast, in the dorsal fin, *actb1* was ranked as the first and *hsp90a* as the second most stable reference genes; except for the SD ranking which ranked *hsp90a* at the top (Table 1). Hence, we used the geometric mean of the expression of *actb1* and *hsp90a* for expression normalization of candidate target genes.

**Table 1:** Ranking and statistical analyses of reference genes in the dorsal and anal fins.

	BestKeeper				geNorm		NormFinder	
	Ranking	SD	Ranking	r	Ranking	M	Ranking	SV
Dorsal fin	*hsp90a*	0.536	*actb1*	0.948	*actb1*	0.465	*actb1*	0.148
	*actb1*	0.609	*hsp90a*	0.941	*hsp90a*	0.469	*hsp90a*	0.168
	*rps18*	0.627	*rps18*	0.931	*rps18*	0.479	*rps18*	0.193
	*rps11*	0.655	*rps11*	0.912	*rps11*	0.505	*rps11*	0.250
	*tbp*	0.675	*hprt1*	0.884	*tbp*	0.579	*tbp*	0.281
	*elf1a*	0.797	*gapdh*	0.849	*hprt1*	0.583	*hprt1*	0.297
	*hprt1*	0.807	*tbp*	0.826	*gapdh*	0.770	*gapdh*	0.445
	*gapdh*	1.053	*elf1a*	0.557	*elf1a*	0.822	*elf1a*	0.567
Anal fin	*hsp90a*	0.467	*hsp90a*	0.938	*hsp90a*	0.483	*hsp90a*	0.113
	*actb1*	0.504	*actb1*	0.908	*actb1*	0.498	*actb1*	0.162
	*rps11*	0.551	*rps11*	0.904	*rps11*	0.504	*rps11*	0.201
	*rps18*	0.618	*hprt1*	0.801	*hprt1*	0.580	*rps18*	0.280
	*hprt1*	0.626	*rps18*	0.798	*rps18*	0.585	*tbp*	0.313
	*tbp*	0.641	*tbp*	0.782	*tbp*	0.597	*hprt1*	0.320
	*elf1a*	0.778	*gapdh*	0.722	*elf1a*	0.831	*gapdh*	0.483
	*gapdh*	1.051	*elf1a*	0.516	*gapdh*	0.918	*elf1a*	0.561

Abbreviations: SD = Standard deviation, r = Pearson product-moment correlation coefficient, SV = stability value, M = M value of stability.

### Expression analysis of the gene regulatory network

**Figure 3:**
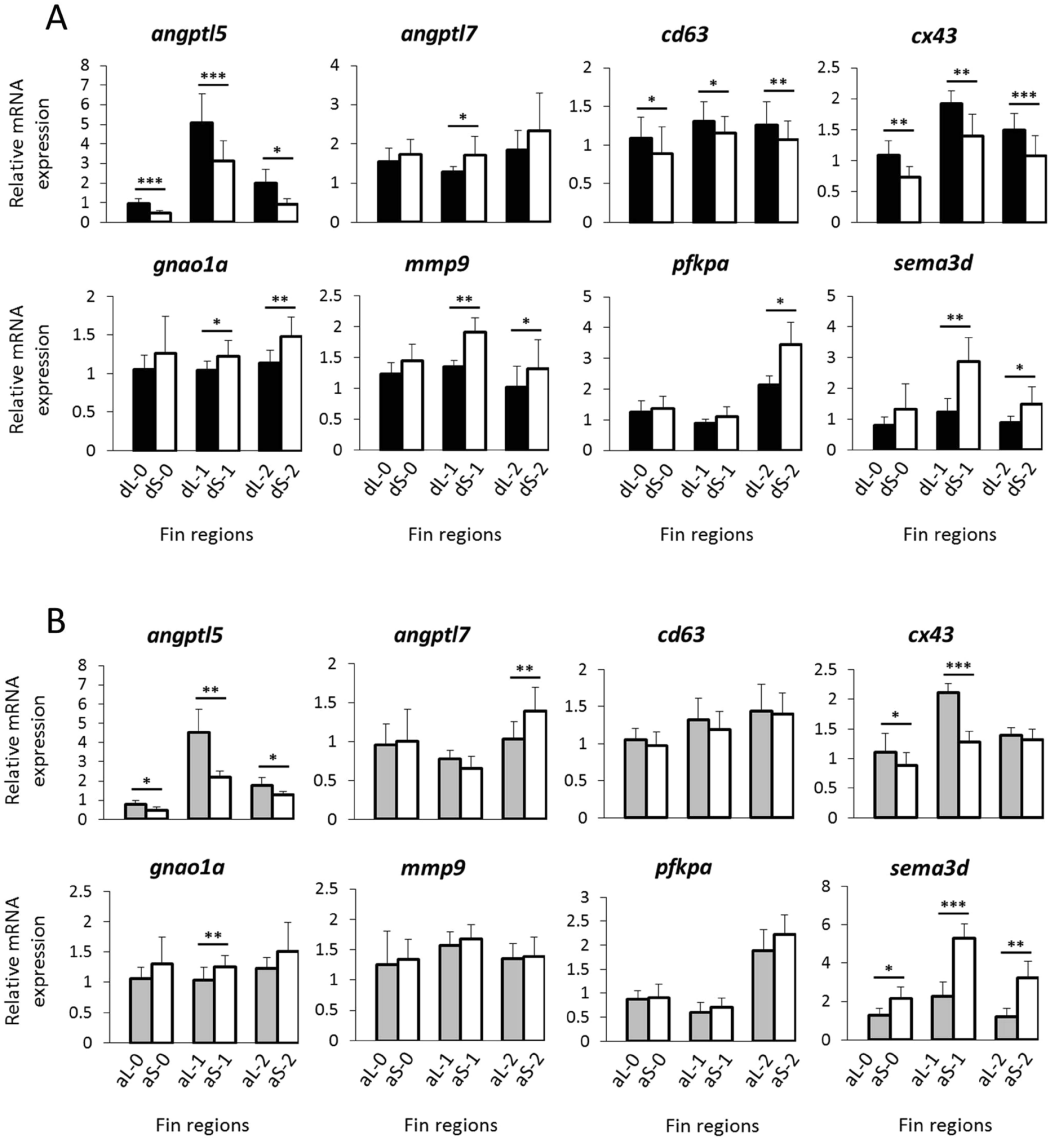
Expression levels of members of a gene regulatory network involved in fin formation/regeneration in the elongated and short fin regions. (**A**) Dorsal fin, (**B**) anal fin. Only genes with significant L/S expression in at least on fin type are included. Means and standard deviations of RQ values in six biological replicates are shown for the elongated (L) and short (S) fin regions; numbers 0 to 2 identify regeneration stages. See Fig. 1A for fin region codes. Significant differences between L and S regions are indicated by asterisks (*P < 0.05; **P < 0.01; ***P < 0.001).

**Figure 4:**
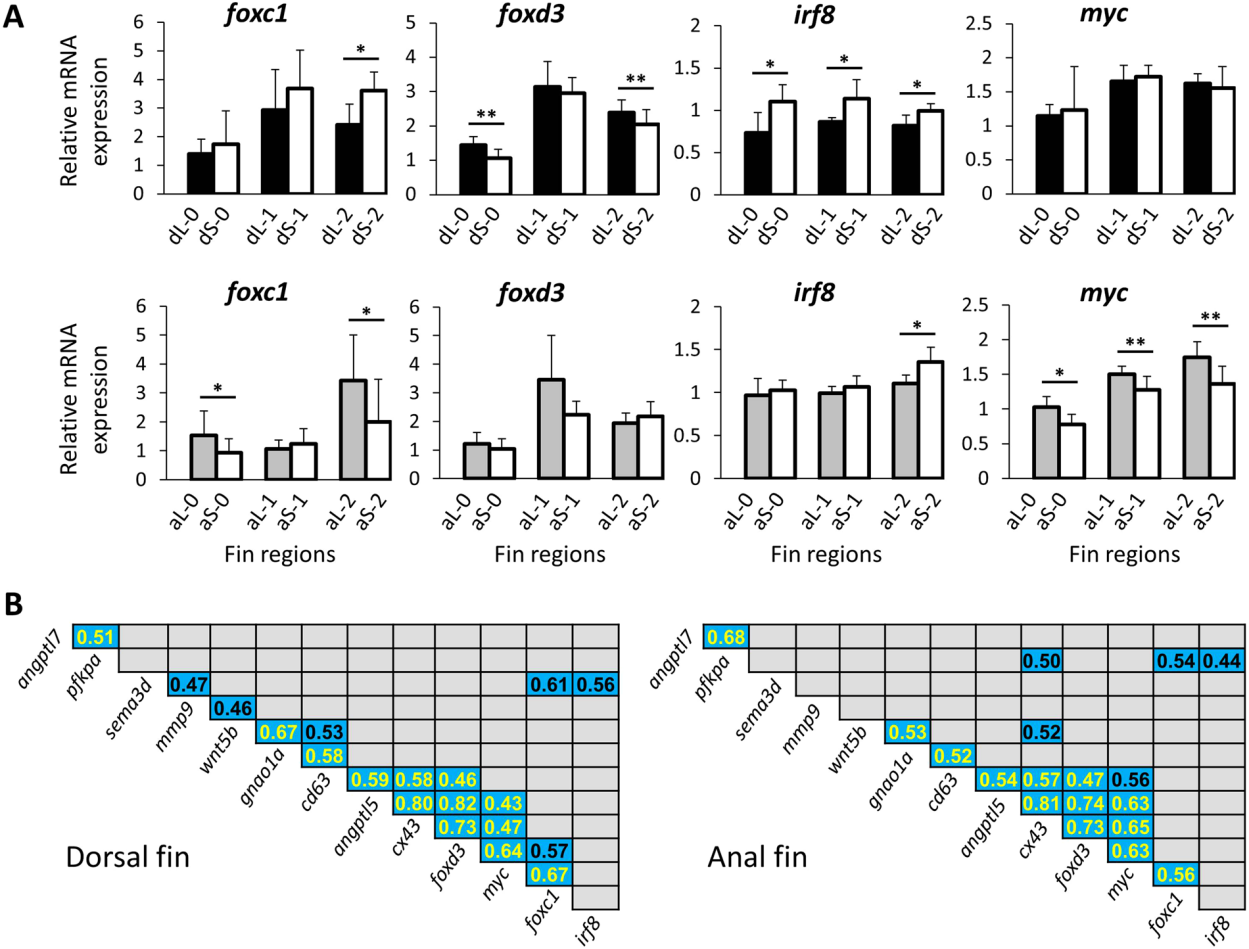
Expression levels of potential regulators of the gene network and pairwise expression correlations. (**A**) Expression levels of upstream regulators of the tested gene regulatory network in the elongated and short region of the dorsal and anal fin. See Fig. 3 for explanations. (**B**) Expression correlations between components of the GRN in the dorsal and anal fins of *S. casuarius*. Numbers show Pearson correlation coefficients that were significant at P < 0.01 and blue shadings represent positive expression correlation (no negative correlations were identified). Yellow font indicates significant expression correlations shared across both fin types.

We tested members of a proposed gene regulatory network associated with fin elongation of the cichlid *Neolamprologus brichardi* (tribe Lamprologini)^16^ for differential expression between elongated (L) and short (S) fin regions in the distantly related cichlid, *Steatocranus casuarius*. The 20 target genes (Table 2) include 12 co-expressed genes with a role in skeletogenesis, angiogenesis and neurogenesis in regenerating tissues; one ligand of the Wnt pathway, *wnt5b*; as well as 6 upstream regulators (transcription factors/TFs) of the network found in *N. brichardi*^16^. Additionally, we tested *esco2*, which is an upstream regulator of *cx43* and *sema3d* in zebrafish^33,34^. Gene expression levels were compared between L and S regions within each fin type (thereby avoiding potentially non-homologous comparisons between anal and dorsal fins)^9^ at three regeneration stages. Stage 0 represents the original fin with expression patterns required for the maintenance of the phenotype, while stage 1 and 2 represent regenerating fin tissue. At stage 1 (20 days of regeneration), fin ray elongation was starting to become apparent, while at stage 2 (40 days of regeneration), the length difference between L and S regions was already obvious but not yet fully recovered (Fig. 1B). After correcting for multiple testing (N = 40 L/S comparisons; 20 genes in 2 fin types), linear mixed models (LMM) identified 13 genes with significant L/S expression differences in one or both fin types (anal and/or dorsal; Supplementary Data 1). We then used post-hoc tests (paired t-tests) to compare L/S expression of these genes separately at each developmental stage (Supplementary Data 1). For one of the 13 genes (*anxa2a*), L/S expression did not differ at any of the individual developmental stages, which reduced our focal gene set to 12 genes (Figs 3 and 4; Supplementary Data 1). ‘Expression in the elongated region’ is abbreviated as ‘L-expression’ in the following text, and reported as ‘higher’ or ‘lower’ in comparison to expression in the short region (‘S-expression’). In most cases when a gene displayed multiple significant L/S expression differences, the differences were in the same direction, i.e. either higher or lower L-expression in different fins or developmental stages (Figs 3 and 4; Supplementary Data 1).

**Table 2:** Expression patterns of the tested genes in *N. brichardi* and the dorsal and anal fins of *S. casuarius*.

gene	*N. brichardi*	*S. casuarius*, dorsal fin	*S. casuarius*, anal fin	Position in the GRN*
*angptl5*	L	L	L	CNM
*angptl7*	L	S	—	CNM
*anxa2a*	L	—	—	CNM
*c1qtnf5*	L	—	—	CNM
*cd63*	L	L	—	CNM
*csrp1a*	L	-	—	CNM
*cx43*	L	L	L	CNM
*dpysl5a*	L	—	—	CNM
*egr2*	L	—	—	UR
*esco2*	L	—	—	UR
*foxc1*	S	—	L	UR
*foxd3*	L	L	—	UR
*foxp1*	S	—	—	UR
*gnao1a*	L	S	—	CNM
*irf8*	L	S	—	UR
*mmp9*	L	S	—	CNM
*myc*	L	—	L	UR
*pfkpa*	L	S	—	CNM
*sema3d*	S	S	S	CNM
*wnt5b*	S	—	—	UR

L: higher expression in the elongated than in the short region of the fins; S: higher expression in the short than in the elongated region of the fins. Dashes indicate that gene expression did not differ significantly between elongated and short fin regions. CNM: coexpression network member; UR: predicted upstream regulator. *The GRN is predicted based on our previous study of *N. brichardi*.

The number of genes with L/S expression differences according to the above analyses was higher in the dorsal fin (N = 10 genes) than in the anal fin (N = 5 genes; Table 2). In the dorsal fin, higher L-expression was detected for *angptl5*, *cd63*, *cx43* and *foxd3*, while lower L-expression was detected for *angptl7*, *gnao1a*, *irf8*, *mmp9*, *pfkpa* and *sema3d*. In the anal fin, congruent results were obtained for *angptl5* and *cx43* (higher L-expression) as well as for *sema3d* (lower L-expression). Additionally, *foxc1* and *myc* showed higher L-expression in the anal fin (but not in the dorsal fin). Note that a significant result in the LMM did not necessarily imply significant L/S expression differences at each of the individual developmental stages; vice versa, genes that showed a significant L/S expression difference at only one developmental stage were not necessarily picked up by the LMM analyses. Among the 12 focal genes, only *angptl5* showed significant L/S expression differences (higher L-expression) in both fin types and at all developmental stages. For *cx43*, significantly higher L-expression was detected in all contrasts except at stage 2 in the anal fin. These results suggest that differential L/S expression of these genes may be involved in both maintenance and regeneration of the anal and dorsal fin elongations (Fig. 3). Six of the genes showed differential L/S expression at stage 0 in either the anal fin (*sema3d*, *foxc, myc*) or the dorsal fin (*cd63*, *foxd3*, *irf8*), which may reflect fin-specific roles of these genes in the maintenance of the fin phenotypes. In contrast, differential L/S expression of *angptl7* and *gnao1a* (anal and dorsal fin), and *mmp9* (dorsal fin) occurred only during regeneration and may not be required for the maintenance of the elongated fin phenotypes in the original fin (stage 0) (Fig. 3).

### Fin specific expression patterns

**Figure 5:**
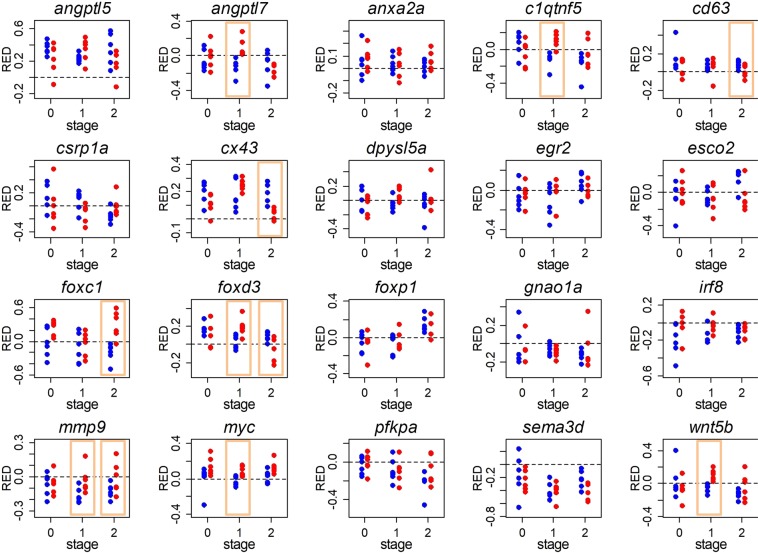
Comparisons of L/S expression patterns between fins. For each of the tested genes, relative expression differences between L and S tissue (RED values) are shown at each regeneration stage and for each fin type (blue dots: dorsal fin; red dots: anal fin). Each dot represents one biological replicate. RED > 0 indicates higher expression in L than in S tissue (L > S expression), while RED < 0 indicates L < S expression. Yellow squares highlight significant differences in RED values between dorsal and anal fins (Mann-Whitney U-test, p < 0.05).

The above comparisons of L- versus S-expression in the dorsal and anal fins suggested fin-specific expression patterns for several of the investigated genes. To further explore this, we calculated relative L/S expression differences as RED = (RQ_L_ − RQ_S_)/(RQ_L_ + RQ_S_) for each of the 20 network genes in each fin type separately, and then used paired Mann-Whitney U-tests to compare RED values between fin types (Fig. 5). Significant differences between fin types were detected only during regeneration (developmental stage 1 and/or 2). Most interestingly, RED values of *mmp9* differed significantly between anal and dorsal fins at both stage 1 and stage 2, which confirms that the differential regulation of *mmp9* during the regeneration of the L versus the S tissue is specific to the dorsal fin. Comparisons of RED values between fin types also confirmed fin-specific L/S expression patterns (with significant L/S differences detected in only one fin type) of *angptl7*, *cd63*, *foxc1*, *foxd3* and *myc*. Additionally, *cx43* had shown L > S expression in both fins in the LMM analysis, and the significant RED differences between fin types during late regeneration (stage 2) indicate that L/S differences persist longer in the dorsal than in the anal fin. Finally, RED values of *wnt5b* differed significantly between fin types during early regeneration (stage 1), although neither fin type had shown significant L/S differences in the LMM analyses. While we note that stage-specific differences between fins may also be influenced by possibly asynchronous regeneration of the two fin types, our observations suggest that the different phenotypes of the anal and dorsal fins (moderate versus distinct elongation) are associated with fin-specific expression patterns of some of the tested network genes.

### Expression correlations between members of the gene network

In our previous study on fin shape formation in the Princess cichlid *N. brichardi*, we analysed gene expression correlations to identify potential members of the gene regulatory network that controls fin elongations. Expression levels of many of the putative GRN genes, which were now tested in *S. casuarius*, were positively correlated in the *N. brichardi* fins; only *sema3d* expression was negatively correlated with some of the network members and potential upstream regulators^16^. In contrast, the analyses of pairwise expression correlations among genes with significant L/S differences in *S. casuarius* yielded a comparatively small number of significant results (Fig. 5B; for all 20 genes, see Supplementary Fig. 1), most of which indicated positive correlations. The majority of the expression correlations were replicated across both fins. For instance, the positive expression correlations between *angptl5-cx43-foxd3-myc* suggest conserved regulatory connections between the regulatory factors *foxd3* and *myc* and their potential downstream effectors *angptl5* and *cx43*.

A noteworthy exception was the expression of *sema3d*, which was positively correlated with *mmp9*, *foxc1* and *irf8* in the dorsal fin, whereas it was not correlated with any of the 12 focal genes in the anal fin (Fig. 5B), suggesting fin-specific and *foxc1-irf8*-dependent transcriptional regulation of *sema3d*. The expression of *pfkpa* was positively correlated with *cd63*, *foxc1* and *irf8* in the anal fin, but not in the dorsal fin, and expression of *cd63* was positively correlated with *myc* in the anal fin only (Fig. 5B), possibly implying differences in the transcriptional regulation of *cd63* and *pfkpa* between the two fin types.

## Discussion

In our previous study of fin elongation in the Princess cichlid, *Neolamprologus brichardi*, we used qPCR-based expression profiling of candidate genes to identify potential members of a gene regulatory network (GRN) involved in fin formation and/or regeneration during both maintenance and regeneration stages^16^. The proposed GRN included some functionally characterized members such as *cx43*, *sema3d* and *mmp9*, which are known to be involved in the formation and growth of fin ray segments in zebrafish^17–20^. Expression patterns in *N. brichardi* supported a scenario in which *foxd3* acts as transcriptional activator of *cx43* and *mmp9* (along with a suite of other genes) and repressor of *sema3d*^16^. Here, we investigated whether the same GRN might be involved in the formation of a similar phenotype, i.e. anal and dorsal fin elongation, in another cichlid fish, *S. casuarius*. Fin elongation in *S. casuarius* is far more pronounced in the dorsal fin than in the anal fin, which furthermore allowed us to examine whether gene expression patterns in the two fin types reflect the different elongation phenotypes of the anal and dorsal fin.

Gene expression patterns in *S. casuarius* contrasted with predictions based on the patterns observed in *N. brichardi*. Regarding the links between *foxd3* and *cx43*, *mmp9* and *sema3d*, we found that while L/S expression differences of *cx43* and *mmp9* were congruent in *N. brichardi* (L > S), L-expression was higher for *cx43* but lower for *mmp9* in *S. casuarius* fins. Unlike in *N. brichardi*, no expression correlation was found between *foxd3* and *mmp9*, nor between *foxd3* and *sema3d*. Furthermore, while all of the 20 investigated genes had shown significant L/S expression differences in *N. brichardi*, only a subset of these genes showed significant L/S expression differences in *S. casuarius*, some of which were in the opposite direction than in *N. brichardi* (Table 2). Finally, patterns of pairwise gene expression correlation differed markedly between the two species across the 20 investigated genes, with a much smaller of number of correlated genes detected in *S. casuarius* than in *N. brichardi*. We note that differences in the results of the two studies, *S. casuarius* versus *N. brichardi*, could be related to their relative power to detect significant gene expression patterns, given that the sampling regime differed between species. In *N. brichardi*, gene expression was measured in three biological replicates, each of which combined corresponding tissue samples from eight fish. In contrast, in *S. casuarius*, each individual fish represented one biological replicate (n = 6 replicates). Pooling individual tissues into biological replicates is expected to reduce the variation among replicates, and indeed, coefficients of variation (CV per gene, fin type, developmental stage and fin region) were substantially lower in *N. brichardi* (mean CV ± sd = 0.13 ± 0.08) than in *S. casuarius* (mean CV ± sd = 0.26 ± 0.12; t = 14.8, p < 0.0001). However, we found no relationship between the coefficients of variation for individual genes and the failure or success to detect significant L/S expression differences and expression correlations in *S. casuarius*. Therefore, while non-significant contrasts and correlations do not necessarily imply the absence of expression differences and correlations in *S. casuarius*, there is no indication that the different experimental designs systematically affected the comparison between the two studies.

In *N. brichardi*, expression patterns of some GRN genes did not conform to inferences on their regulatory interactions in zebrafish^16^, whereas expression patterns of some of these genes in *S. casuarius* are consistent with the zebrafish data (Supplementary Fig. 2). For instance, in the zebrafish, reduced expression of *cx43* was associated with up-regulation of *mmp9*^18^. In *N. brichardi*, higher L-expression of both *cx43* and *mmp9* contradicted the zebrafish pattern, whereas the two genes showed the expected opposite L/S expression differences in the dorsal fin of *S. casuarius*. Similarly, *esco2* is known as upstream transcriptional regulator of *cx43* and *sema3d* in the zebrafish^33,34^. Whereas the expression of *esco2* was not correlated with *cx43* and *sema3d* in *N. brichardi*, positive expression correlations of both genes with *esco2* were detected in both fins of *S. casuarius* (Supplementary Fig. 1). An unexpected finding, compared to zebrafish data and expression patterns in *N. brichardi*, is the expression pattern of the transcription factor *irf8*. In zebrafish larvae, *irf8* is required for cell proliferation and fin regeneration^26^ and L > S expression of *irf8* in *N. brichardi* was consistent with this function^16^. In *S. casuarius*, however, the elongated region of the dorsal fin showed significantly lower *irf8* expression than the short region, which contrasts with the predicted positive correlation between *irf8* expression and fin outgrowth. Finally, *S. casuarius* and *N. brichardi* share a discrepancy with the zebrafish model in that we detected no correlations between the expression levels of *cx43* and *sema3d*, although *sema3d* acts downstream of *cx43* in zebrafish^17^.

Although superficially similar, we found that the fin elongation phenotype differs anatomically between *S. casuarius* and *N. brichardi*. In *N. brichardi*, segments of the elongated fin rays are not longer than in the short rays, and fin ray elongation is therefore due to increased segment number^16^. This was surprising given the significant L > S expression of *cx43*, as *cx43* expression is positively related with segment growth in zebrafish^17–20^. In contrast, data from *S. casuarius* are consistent with the function of *cx43* in zebrafish, as elongated fin regions had both higher *cx43* expression *and* longer fin ray segments than the short fin regions (Fig. 1C,D, Supplementary Table 1, Fig. 3).

The differences between the two cichlid species in relation to the zebrafish model are interesting in a phylogenetic context. *N. brichardi* originated within the Lake Tanganyika radiation as a member of the tribe Lamprologini, while the Steatocranini (the cichlid tribe holding the genus *Steatocranus*) are a sister lineage to the Lake Tanganyika cichlids and the other Great Lakes radiations^29^. It now remains to be tested whether the gene functions and GRN composition observed in *N. brichardi* are unique to this species, shared by other Lamprologini (some of which sport elongated fins) or even across the large East African lacustrine cichlid radiations, or whether perhaps *S. casuarius* regained some of the zebrafish-like gene functions independently. Remarkably, in a comparative analysis of the evolution of regulatory elements across African cichlid species representing the Great Lakes radiations, the number of ‘accelerated conserved noncoding elements’ (CNEs evolving at a significantly accelerated rate) was highest in *N. brichardi*^35^. Before this background, it may be hypothesized that between-species differences observed in our study are due to the accelerated regulatory evolution in the lineage hosting *N. brichardi*.

The different phenotypes of the dorsal and the anal fins of *S. casuarius* coincided with differences in gene expression patterns. The dorsal fin displays a more pronounced elongation and a larger difference in fin ray segment lengths between elongated and short fin regions than the anal fin (Fig. 1). Accordingly, a larger number of the investigated genes showed significant L/S expression differences in the dorsal fin than in the anal fin. One of them is *cd63*, which induces spinal cord regeneration in the axolotl^24^. Notably, proper innervation of fin rays has already been demonstrated to be essential not only for maintenance of proliferative blastemal cells during fin regeneration but also for tissue morphogenesis during fin outgrowth^36^. Hence, the dorsal fin-specific L > S expression in *S. casuarius* might reflect more active neural regeneration in the distinctly elongated region of the dorsal fin than in the moderately elongated anal fin, although L > S expression at stage 0 also indicates a role in innervation maintenance. Differences in the L/S expression patterns of *cx43*, *mmp9* and *foxd3* between the two fin types are particularly interesting because of their proposed regulatory interactions and their roles in fin ray growth^11,16,18–20^. In zebrafish, the decreased expression of *cx43* leads to up-regulation of *mmp9* in the caudal fin^18^ and to defects in the lengthening of bony fin ray segments^19^. In *S. casuarius*, *cx43* showed significantly L > S expression in both fin types, but the expected converse pattern of *mmp9* (i.e. L < S expression) was observed only in the dorsal fin, where the difference in segment length between elongated and short fin rays was more pronounced than in the anal fin. Similarly, the transcription factor *foxd3*, which was associated with the exaggerated fin outgrowth (the sword) of male sword-tail fish^11^ and proposed as a main upstream regulator of the fin elongation GRN in *N. brichardi*^16^, showed significant L > S expression only in the dorsal fin of *S. casuarius*. In zebrafish, *foxd3* is implicated in the dedifferentiation associated with tissue regeneration through its balancing effects on BMP and Wnt signals^37,38^. Together, these data support the hypothesis that expression of *foxd3* promotes fin outgrowth in different fish species; however, the GRN comparison between *S. casuarius* and *N. brichardi* suggests that it may do so via different pathways. Likewise, divergent expression patterns of the transcription factors *irf8* (see above) and *foxc1* (higher and lower L-expression in *S. casuarius* and *N. brichardi*, respectively) hint at variation in the regulatory pathways between species. Interestingly, however, positive correlations between *sema3d* and *foxc1* were identified in both *S. casuarius* (dorsal fin only) and *N. brichardi*, indicating potential regulation of *sema3d* by *foxc1* in both cichlid species. Whereas the majority of pairwise gene expression correlations were detected in both the anal and dorsal fin of *S. casuarius*, the small number of fin-specific correlations, including the one between *sema3d* and *foxc1*, may indicate regulatory differentiation between the two fin types, although non-significant correlations may also be due to small sample sizes.

Finally, the fin-specific expression pattern of the transcription factor *myc* was puzzling with regard to the function of this gene in other animals. *myc* encodes a nuclear phosphoprotein which can induce pluripotency in differentiated cell types in vertebrates^39^ and its expression is elevated in blastemal cells of regenerating *Xenopus* tails^25^. Accordingly, *myc* expression was higher in the elongated fin regions of *N. brichardi* than in the short regions^16^. Surprisingly, this was not the case in the distinctly elongated dorsal fin of *S. casuarius*, whereas the moderately elongated anal fin showed the expected L > S expression difference. This confounding result might be partially explained by complex auto-regulatory characteristics of *myc*, through which negative feedback loops limit its function^40–43^. In addition, it has been shown that prolonged expression of *myc* in murine cells can lead to premature induction of apoptosis rather than stimulate cell proliferation^44^. Similarly, another study has shown that activation of the canonical WNT pathway together with overexpression of *myc* induces a proliferative status in cancer cells, whereas prolonged overexpression of *myc* alone leads to apoptosis^45^. The modulation of the balance between *myc*-dependent cell proliferation/growth and apoptosis in different cell types and physiological conditions has been reviewed in mammalian cells^46,47^. Taken together, these observations propose a model in which the increased L-expression of *myc* during early fin formation can induce a proliferative status and subsequently slight elongation in the L region of the anal fin of *S. casuarius*. Later, its prolonged expression may have self-limiting effects through increased apoptosis which in turn constrains the elongation process in the anal fin.

## Conclusions

In a previous study, we proposed 20 genes as putative members and regulators of a GRN associated with the formation and regeneration of ornamental fin elongations in the cichlid fish *N. brichardi*. Here, we found that some of those genes, particularly *cx43, mmp9* and *sema3d*, are also differentially expressed in the elongated fins of another cichlid species, *S. casuarius*. However, despite the superficial similarity of fin shape in the two species, the anatomical basis of the elongations as well as the gene expression patterns of most (75%) candidate genes and transcription regulators differed between *N. brichardi* and *S. casuarius*. While this suggests that there is considerable regulatory divergence between the two cichlid species with regard to the formation of their convergent fin phenotypes, we emphasize that not all of the observed expression differences between the two cichlid species are necessarily linked to differential regulation of fin elongation. The members of the GRN were proposed based on qPCR expression profiling in *N. brichardi*, but were not validated for their involvement with fin elongation by functional analyses. Therefore, although the candidate genes tested in *N. brichardi* had been carefully selected based on their roles in fin growth and regeneration, some of them may display L/S expression difference in a context other than control of fin shape. This needs to be investigated by future work. The fact that we find anatomical differences with respect to how elongation is achieved in the two species, however, predicts that the underlying genetic mechanisms should also differ between them. Fin elongations evolved several times independently within Cichlidae, providing an excellent phylogenetic background for further comparative analyses. According to current evidence, regulatory divergence between species is mainly driven by *cis* evolution^3,48^, which suggests that the molecular basis of the observed differences may be identified in the upstream regions of the differentially regulated genes.

## Methods

### Sampling and characterization of the fins

Six captive bred adult males of *S. casuarius* with total lengths of 10–12 cm were used to conduct the experiment. The fish were kept in one large tank (approx. 500 L) together with a group of 18 females. Each male was allocated a shelter consisting of a cluster of two to three PVC plastic tubes. Males stayed near their shelters most of the time and showed little movement and no aggression. Prior to taking the fin biopsies, fish were anesthetized in a solution of 0.04 g MS–222per 1 L water. To obtain tissue for gene expression analyses, the dorsal and anal fins were cut anterior to the first bifurcation of the fin rays (most proximal branching, see red lines in Fig. 1A,B) under a stereomicroscope. Next, two tissue samples were cut from this biopsy, one representing the elongated (L) and the other the short (S) fin region. These samples were cut immediately distal to the bifurcation and included the two longest rays of each fin for the L sample and the second- and third-shortest ray of each fin for the S sample (Fig. 1A; the very shortest ray was avoided because it was only rudimentarily developed in some fins). Hence, each tissue sample consisted of fin rays and inter-ray tissue between the most proximal ray bifurcation and the distal fin edge. The tissue biopsies were immersed in RNAlater (Qiagen) and stored at −20 C°. Following this scheme, biopsies were taken from the intact fins to represent the maintenance stage (stage 0) and during regeneration. We therefore performed another biopsy 20 days after the first cut. At this early regeneration stage (stage 1), the elongations had just become apparent in both fin types, but the difference between the anal and the dorsal fin had not yet developed (Fig. 1B). Finally, a third biopsy was taken 40 days after the second cut. At this late regeneration stage (stage 2), the elongations were pronounced but still incomplete and the difference in the extent of elongation between anal and dorsal fins was already visible (Fig. 1B).

To measure the length of fin ray segments in elongated and short regions, fin tissue samples were taken from five adult males and stained with alizarin red. We modified the acid-free double staining protocol described by^49^ and used 10% KOH in the clearing phase, increased the duration of the staining phase to 4 days and the duration of the clearing phase to 15 days. Using a Keyence VHX-5000 Digital Microscope, we measured the lengths of the 10 most distal, complete segments of two long and two short fin rays. For the elongated region, we measured segment lengths on one of the two branches of the two longest fin rays (i.e. measures were taken from two different rays, not from branches of the same ray). The branch was either selected randomly or by avoiding irregularities in the segmentation pattern, which sometimes occurred in one of the branches. Using the same criteria, we selected one branch of the second-shortest ray and one of the third-shortest ray for segment length measurements in the short fin region. The very shortest ray was avoided because it was only rudimentarily developed in some fins.

Anaesthesia and fin biopsies were performed under permit number BMWFWF-66.007/0028-WF/V/3b/2017 issued by the Federal Ministry of Science, Research and Economy of Austria in accordance with the guidelines and regulations of BMWFW.

### RNA isolation and synthesis of first strand cDNA

RNA was isolated from a total of 72 tissue samples (6 replicates per fin and region and developmental stage) using the ReliaPrep™ RNA Tissue Miniprep System Kit (Promega). Tissue samples were transferred to tubes containing 1.4 mm ceramic spheres and 250 µl of LBA buffer mixed with the recommended volume of 1-Thioglycerol. The tissues were homogenized using FastPrep-24 Instrument (MP Biomedicals, CA, USA) and RNA isolation steps were performed according to manufacturer’s ReliaPrep™ protocol for RNA extraction from fibrous tissues. The ReliaPrep™ protocol includes genomic DNA removal (DNase treatment). The purification steps are conducted via a filtered column without the use of phenol-chloroform extractions or ethanol precipitations. The protocol is well-adjusted to isolate high quality RNA from small amounts of fibrous tissues, and allowed us to obtain a sufficient amount of RNA from each of the small tissue samples. After eluting the isolated RNA in 25 µl nuclease-free water, we measured the concentrations with a Nanophotometer (IMPLEN GmbH, Munich, Germany). The quality of 20 randomly selected samples was checked in a R6K ScreenTape System on an Agilent 2200 TapeStation (Agilent Technologies) and exceeded 8 RIN (RNA integrity number) in all samples. The first strand cDNA was synthesized from 400 ng of RNA by following the manufacturer’s protocol supplied with the High Capacity cDNA Reverse Transcription kit (Applied Biosystems) and cDNA was diluted 1:3 times in nuclease-free water as input for qPCR.

### RNA-Seq and *de novo* transcriptome assembly

To obtain a comprehensive list of transcripts from *S. casuarius* fins, we selected one sample with a high RNA integrity number from each fin type and then pooled together with equal quantity for transcriptome sequencing. The library preparation was performed using a Standard TruSeq Stranded mRNA Sample Prep Kit (Illumina), following the manufacturer’s protocol, with an RNA input of 1200 ng. Subsequently, the quality of the library preparation was checked using a D1000 ScreenTape and reagents (Agilent) on a TapeStation 2200 (Agilent). Finally, the library was diluted and the sequencing was performed by the NGS Facility at Vienna Biocenter Core Facilities (VBCF, Austria) to produce 125 bp paired-end reads. A quality control of the raw reads was performed using the bioinformatic tool FASTQC^50^. The removal of low quality reads and the quality trimming of the raw reads was carried out using the software Trimmomatic^51^. Finally, the transcriptome of *S. casuarius* was assembled *de novo*, based on the quality trimmed paired-end reads, using the Trinity software package^52,53^. The generated sequence read archives and assembly are available at NCBI under SRA Accession No. SUB4649276.

### Candidate genes, designing primers and qPCR

To identify stable reference gene gene(s), we selected 8 candidate reference genes with abundant expression in a range of tissues, which have already been investigated as reference genes in fins or other tissues containing skeletal structures or/and epidermis in fish (Supplementary Data 2)^14,15,54–56^. As target genes, we selected 20 members of a gene regulatory network involved in fin and tissue morphogenesis and/or regeneration (Supplementary Data 2)^16^. In order to design qPCR primers, we aligned the assembled sequence of each gene obtained from the RNA-Seq data against homologous sequences from different African cichlid tribes including three species in Tilapiini tribe (*O. aureus*, *O. mossambicus* and *O. niloticus*), one species in Ectodini tribe (*Callochromis macrops*), one species in Lamprologini tribe (*Neolamprologus brichardi*) and four species from diverse Haplochromini tribe (*Maylandia zebra*, *Pundamilia nyererei*, *Ctenochromis horeii* and *Astatotilapia burtoni*)^35,57,58^. Next we used the aligned sequences to identify conserved regions across the species (using CLC Genomic Workbench, CLC Bio, Denmark) and at the exon/exon boundaries (using annotated genome of *Oreochromis_niloticus* in the Ensembl database, http://www.ensembl.org). The primers with short amplicon sizes (<250 bp) were designed using Primer Express 3.0 (Applied Biosystems, CA, USA) and their dimerization and secondary structure formation was checked through OligoAnalyzer 3.1 (Integrated DNA Technology) (Supplementary Data 2).

We followed the manufacturer’s protocol for Maxima SYBR Green/ROX qPCR Master Mix (2X) (Thermo Fisher Scientific, Germany) to set up qPCR reactions, which were performed in 96 well-PCR plates on an ABI 7500 real-time PCR System (Applied Biosystems). Each biological replicate was conducted in two technical replicates for each gene and we followed a sample maximization method^59^ to have an optimal experimental set-up in each run. The qPCR was initiated with a 2 min hold at 50 °C followed by a 10 min hold at 95 °C; amplification employed 40 cycles of 15 sec denaturation at 95 °C and 1 min annealing/extension at 60 °C. A dissociation step (60 °C–95 °C) was performed at the end of the amplification step. Finally, we calculated primer efficiencies (E values) using the LinRegPCR v11.0 programme^60^ (Supplementary Data 2).

### Analysis of gene expression data

Candidate reference genes were ranked according to expression stability by three different algorithms, BestKeeper^61^, NormFinder^62^ and geNorm^63^. The standard deviation (SD) based on Cq values of the fin regions was calculated by BestKeeper to determine the expression variation for each reference gene. In addition to ranking, BestKeeper also determines the stability of reference genes through a correlation calculation or BestKeeper index (r). GeNorm calculates mean pairwise variation between each gene and other candidates (the expression stability or *M* value) in a stepwise manner and NormFinder identifies the most stable genes (lowest expression stability values) based on analysis of inter- and intra-group variation in expression levels variations^31^. In this study, the geometric mean of the Cq values^63^ of two most stable reference genes in both fins types, Cq _reference_, was used to normalize Cq values of target genes in each sample (ΔCq _target_ = Cq _target_ − Cq _reference_). In order to calculate ΔΔCq values, we randomly selected one biological replicate of the elongated fin region of the anal and the dorsal fin (i.e. each fin type had its own calibrator sample), and subtracted the ΔCq from the calibrator ΔCq value (ΔCq _target_ − ΔCq _calibrator_). Relative expression quantities (RQ values) were calculated as 2^−ΔΔCq^ ^64^.

RQ values were log-transformed for statistical analyses. For each target gene, differences in expression levels between elongated (L) and short (S) regions of the anal and the dorsal fin were tested in linear mixed models with log(RQ) as dependent variable, length (L or S) as fixed factor and developmental stage nested within biological replicate as grouping factor (Supplementary Data 2). To account for multiple testing (N = 40 L/S comparisons; 20 genes times 2 fin types), p-values for the effect of length were corrected using the Benjamini-Hochberg procedure^65^. Next, we compared L/S expression at each developmental stage of the anal and dorsal fin using paired t-tests (paired by biological replicate). Similarities in expression patterns between the target genes across tissues and developmental stages were examined by calculating pairwise Pearson correlation coefficients.

In order to compare the L/S expression *differences* of individual genes between the anal and the dorsal fin, we first calculated the relative L/S expression difference for each fin type as RED = (RQ_L_ − RQ_S_)/(RQ_L_ + RQ_S_); i.e. corrected for differences in the absolute expression levels between fins. This yielded, for each gene, six RED values for the anal fin and six RED values for the dorsal fin (with N = 6 biological replicates), which were then compared using paired Mann-Whitney U-tests (paired by biological replicate).

To compare fin ray segment length across rays and fin types, we used linear mixed models with segment length as dependent variable, ray or fin type as fixed factor (for comparisons within and across fin type, respectively), and the sampled fish as random factor.

### Ethical approval

All experimental protocols related to the fishes used in this study were approved by the Federal Ministry of Science, Research and Economy of Austria, permit numbers

## Supplementary information

<b>Supplementary Figures</b>

<b>Supplementary Table 1</b>

<b>Dataset 1</b>

<b>Dataset 2</b>

## Data Availability

All the data represented in this study are provided within the main manuscript or in the supplementary materials.
