# Supplementary material for: Gene expression profiling suggests differences in molecular mechanisms of fin elongation between cichlid species
Source: Sci Rep. 2019 Jun 21;9:9052. doi: 10.1038/s41598-019-45599-w (PMC6588699; doi:10.1038/s41598-019-45599-w)
Supplement: Supplementary file 1 — Supplementary Figures [file 41598_2019_45599_MOESM1_ESM.pdf]

Gene expression profiling suggests differences in molecular mechanisms of fin elongation between cichlid species

Ehsan Pashay Ahi[1,2],
Email: ehsanpashayahi@gmail.com

Florian Richter[1],
Email : florianrichter1@outlook.com

Laurène Alicia Lecaudey[1],
Email: laurenelecaudey@gmail.com

Kristina M. Sefc[1],
Email: kristina.sefc@uni-graz.at

1. Institute of Biology, University of Graz, Universitätsplatz 2, A-8010 Graz, Austria

2. Department of Comparative Physiology, Uppsala University, Norbyvägen 18A, SE-75 236 Uppsala, Sweden

Corresponding Author: Ehsan Pashay Ahi,
Email: ehsanpashayahi@gmail.com

angptl5	p= 0.148<br>r= -0.25	p= 0.513<br>r= -0.11	p= 0.901<br>r= -0.021	p<0.001<br>r= 0.59	p= 0.101<br>r= -0.28	p<0.001<br>r= 0.80	p= 0.579<br>r= 0.096	p= 0.043<br>r= -0.34	p<0.001<br>r= 0.76	p= 0.086<br>r= 0.29	p<0.001<br>r= 0.82	p= 0.445<br>r= 0.13	p= 0.831<br>r= 0.037	p= 0.747<br>r= -0.056	p= 0.827<br>r= 0.038	p= 0.009<br>r= 0.43	p= 0.029<br>r= -0.36	p= 0.204<br>r= 0.22	p= 0.208<br>r= 0.22
p= 0.185<br>r= -0.23	angptl7	p= 0.004<br>r= 0.47	p= 0.094<br>r= 0.28	p= 0.673<br>r= 0.073	p<0.001<br>r= 0.56	p= 0.678<br>r= 0.072	p= 0.002<br>r= 0.50	p= 0.003<br>r= 0.48	p= 0.191<br>r= -0.22	p= 0.02<br>r= 0.39	p= 0.841<br>r= -0.035	p= 0.072<br>r= -0.30	p= 0.039<br>r= 0.35	p= 0.348<br>r= 0.16	p= 0.077<br>r= 0.30	p= 0.136<br>r= 0.25	p= 0.001<br>r= 0.51	p= 0.493<br>r= 0.12	p= 0.138<br>r= 0.25
p= 0.632<br>r= 0.083	p<0.001<br>r= 0.62	anxa2a	p= 0.288<br>r= -0.18	p= 0.002<br>r= 0.49	p= 0.06<br>r= 0.32	p= 0.545<br>r= 0.10	p= 0.077<br>r= 0.30	p= 0.799<br>r= 0.044	p= 0.062<br>r= -0.31	p= 0.837<br>r= -0.036	p= 0.87<br>r= -0.028	p= 0.207<br>r= -0.22	p= 0.001<br>r= 0.52	p= 0.21<br>r= -0.21	p= 0.094<br>r= -0.28	p= 0.184<br>r= -0.23	p= 0.003<br>r= 0.49	p= 0.196<br>r= -0.22	p= 0.113<br>r= 0.27
p= 0.254<br>r= 0.19	p= 0.104<br>r= 0.28	p= 0.596<br>r= 0.092	c1qtnf5	p= 0.534<br>r= -0.11	p= 0.874<br>r= -0.027	p= 0.72<br>r= 0.062	p<0.001<br>r= 0.55	p= 0.047<br>r= 0.33	p= 0.853<br>r= 0.032	p= 0.004<br>r= 0.47	p= 0.1<br>r= 0.28	p= 0.813<br>r= -0.041	p= 0.894<br>r= -0.023	p= 0.639<br>r= 0.081	p= 0.008<br>r= 0.43	p= 0.003<br>r= 0.48	p= 0.202<br>r= 0.22	p= 0.016<br>r= 0.40	p= 0.683<br>r= 0.071
p= 0.001<br>r= 0.54	p= 0.045<br>r= 0.34	p<0.001<br>r= 0.67	p= 0.075<br>r= 0.30	cd63	p= 0.516<br>r= -0.11	p<0.001<br>r= 0.58	p= 0.119<br>r= 0.26	p= 0.304<br>r= -0.18	p= 0.083<br>r= 0.29	p= 0.605<br>r= 0.089	p= 0.004<br>r= 0.46	p= 0.785<br>r= -0.047	p<0.001<br>r= 0.58	p= 0.094<br>r= -0.28	p= 0.469<br>r= -0.12	p= 0.711<br>r= -0.064	p= 0.979<br>r= 0.0046	p= 0.368<br>r= -0.15	p= 0.001<br>r= 0.53
p<0.001<br>r= -0.66	p= 0.008<br>r= 0.44	p= 0.041<br>r= 0.34	p= 0.313<br>r= -0.17	p= 0.731<br>r= -0.059	csrp1a	p= 0.523<br>r= 0.11	p= 0.203<br>r= 0.22	p= 0.008<br>r= 0.44	p= 0.052<br>r= -0.33	p= 0.145<br>r= 0.25	p= 0.485<br>r= -0.12	p= 0.311<br>r= -0.17	p= 0.14<br>r= 0.25	p= 0.443<br>r= -0.13	p= 0.566<br>r= -0.099	p= 0.282<br>r= 0.18	p= 0.001<br>r= 0.52	p= 0.324<br>r= -0.17	p= 0.984<br>r= 0.0034
p<0.001<br>r= 0.81	p= 0.747<br>r= 0.056	p= 0.275<br>r= 0.19	p= 0.005<br>r= 0.46	p<0.001<br>r= 0.57	p= 0.002<br>r= -0.50	cx43	p= 0.016<br>r= 0.40	p= 0.852<br>r= -0.032	p= 0.002<br>r= 0.50	p= 0.034<br>r= 0.35	p<0.001<br>r= 0.73	p= 0.763<br>r= -0.052	p= 0.337<br>r= 0.16	p= 0.122<br>r= -0.26	p= 0.479<br>r= 0.12	p= 0.004<br>r= 0.47	p= 0.196<br>r= -0.22	p= 0.608<br>r= 0.088	p= 0.046<br>r= 0.33
p= 0.983<br>= -0.0036	p= 0.046<br>r= 0.33	p= 0.037<br>r= 0.35	p<0.001<br>r= 0.57	p= 0.043<br>r= 0.34	p= 0.607<br>r= 0.089	p= 0.042<br>r= 0.34	dpysl5a	p= 0.294<br>r= 0.18	p= 0.663<br>r= -0.075	p= 0.073<br>r= 0.30	p= 0.295<br>r= 0.18	p= 0.04<br>r= -0.34	p= 0.033<br>r= 0.36	p= 0.593<br>r= -0.092	p= 0.013<br>r= 0.41	p= 0.163<br>r= 0.24	p= 0.636<br>r= 0.082	p= 0.318<br>r= 0.17	p= 0.005<br>r= 0.46
p<0.001<br>r= -0.65	p= 0.001<br>r= 0.55	p= 0.033<br>r= 0.36	p= 0.387<br>r= 0.15	p= 0.741<br>r= -0.057	p<0.001<br>r= 0.70	p= 0.033<br>r= -0.36	p= 0.041<br>r= 0.34	egr2	p= 0.314<br>r= -0.17	p= 0.063<br>r= 0.31	p= 0.836<br>r= -0.036	p= 0.424<br>r= 0.14	p= 0.555<br>r= 0.10	p= 0.749<br>r= 0.055	p= 0.323<br>r= 0.17	p= 0.335<br>r= 0.17	p= 0.077<br>r= 0.30	p= 0.245<br>r= 0.20	p= 0.943<br>r= -0.012
p<0.001<br>r= 0.78	p= 0.038<br>r= -0.35	p= 0.945<br>r= 0.012	p= 0.414<br>r= 0.14	p= 0.009<br>r= 0.43	p<0.001<br>r= -0.70	p<0.001<br>r= 0.57	p= 0.932<br>r= -0.015	p<0.001<br>r= -0.61	esco2	p= 0.017<br>r= 0.40	p<0.001<br>r= 0.73	p= 0.035<br>r= 0.35	p= 0.542<br>r= -0.11	p= 0.126<br>r= 0.26	p= 0.322<br>r= 0.17	p= 0.001<br>r= 0.52	p= 0.036<br>r= -0.35	p= 0.004<br>r= 0.47	p= 0.901<br>r= 0.021
p= 0.641<br>r= 0.081	p= 0.026<br>r= 0.37	p<0.001<br>r= 0.64	p= 0.104<br>r= 0.28	p= 0.083<br>r= 0.29	p= 0.227<br>r= 0.21	p= 0.277<br>r= 0.19	p= 0.024<br>r= 0.38	p= 0.012<br>r= 0.41	p= 0.807<br>r= -0.042	foxc1	p<0.001<br>r= 0.57	p= 0.12<br>r= 0.26	p= 0.401<br>r= 0.14	p= 0.272<br>r= 0.19	p= 0.073<br>r= 0.30	p<0.001<br>r= 0.67	p= 0.03<br>r= 0.36	p<0.001<br>r= 0.61	p= 0.913<br>r= 0.019
p<0.001<br>r= 0.74	p= 0.908<br>r= -0.02	p= 0.93<br>r= -0.015	p= 0.002<br>r= 0.49	p= 0.004<br>r= 0.47	p<0.001<br>r= -0.57	p<0.001<br>r= 0.73	p= 0.864<br>r= 0.03	p= 0.015<br>r= -0.40	p= 0.001<br>r= 0.53	p= 0.306<br>r= 0.18	foxd3	p= 0.442<br>r= 0.13	p= 0.869<br>r= 0.028	p= 0.874<br>r= -0.027	p= 0.499<br>r= 0.12	p<0.001<br>r= 0.64	p= 0.786<br>r= -0.047	p= 0.031<br>r= 0.36	p= 0.446<br>r= 0.13
p= 0.631<br>r= 0.083	p= 0.058<br>r= 0.32	p= 0.112<br>r= 0.27	p= 0.289<br>r= 0.18	p= 0.155<br>r= 0.24	p= 0.976<br>r= 0.0053	p= 0.679<br>r= -0.071	p= 0.578<br>r= -0.096	p= 0.488<br>r= -0.12	p= 0.986<br>r= 0.0029	p= 0.237<br>r= 0.20	p= 0.177<br>r= 0.23	foxp1	p= 0.428<br>r= -0.14	p= 0.505<br>r= 0.11	p= 0.187<br>r= -0.22	p= 0.266<br>r= 0.19	p= 0.772<br>r= 0.05	p= 0.018<br>r= 0.39	p= 0.002<br>r= -0.51
p= 0.682<br>r= 0.071	p= 0.027<br>r= 0.37	p= 0.003<br>r= 0.49	p= 0.417<br>r= -0.14	p= 0.001<br>r= 0.52	p= 0.153<br>r= 0.24	p= 0.485<br>r= 0.12	p= 0.078<br>r= 0.30	p= 0.372<br>r= 0.15	p= 0.513<br>r= 0.11	p= 0.163<br>r= 0.24	p= 0.801<br>r= 0.044	p= 0.472<br>r= 0.12	gnao1a	p= 0.566<br>r= 0.099	p= 0.709<br>r= 0.064	p= 0.062<br>r= -0.31	p= 0.037<br>r= 0.35	p= 0.62<br>r= 0.085	p<0.001<br>r= 0.67
p= 0.923<br>r= 0.017	p= 0.063<br>r= 0.31	p= 0.021<br>r= 0.38	p= 0.85<br>r= -0.033	p= 0.178<br>r= 0.23	p= 0.268<br>r= 0.19	p= 0.767<br>r= -0.051	p= 0.486<br>r= -0.12	p= 0.535<br>r= 0.11	p= 0.416<br>r= 0.14	p= 0.935<br>r= 0.014	p= 0.844<br>r= 0.034	p= 0.588<br>r= 0.093	p= 0.124<br>r= 0.26	irf8	p= 0.027<br>r= 0.37	p= 0.408<br>r= 0.14	p= 0.922<br>r= 0.017	p<0.001<br>r= 0.56	p= 0.959<br>r= 0.0089
p= 0.2<br>r= 0.22	p= 0.439<br>r= -0.13	p= 0.158<br>r= -0.24	p= 0.001<br>r= 0.54	p= 0.792<br>r= -0.045	p= 0.17<br>r= -0.23	p= 0.016<br>r= 0.40	p= 0.146<br>r= 0.25	p= 0.932<br>r= -0.015	p= 0.151<br>r= 0.24	p= 0.889<br>r= 0.024	p= 0.034<br>r= 0.35	p= 0.55<br>r= -0.10	p= 0.643<br>r= -0.08	p= 0.645<br>r= 0.079	mmp9	p= 0.218<br>r= 0.21	p= 0.158<br>r= -0.24	p= 0.004<br>r= 0.47	p= 0.004<br>r= 0.46
p<0.001<br>r= 0.63	p= 0.961<br>r= 0.0083	p= 0.011<br>r= 0.42	p= 0.005<br>r= 0.46	p<0.001<br>r= 0.56	p= 0.106<br>r= -0.27	p<0.001<br>r= 0.65	p= 0.127<br>r= 0.26	p= 0.578<br>r= -0.096	p= 0.001<br>r= 0.53	p<0.001<br>r= 0.56	p<0.001<br>r= 0.63	p= 0.645<br>r= 0.08	p= 0.875<br>r= 0.027	p= 0.227<br>r= 0.21	p= 0.073<br>r= 0.30	myc	p= 0.4<br>r= 0.14	p= 0.017<br>r= 0.40	p= 0.106<br>r= -0.27
p= 0.398<br>r= -0.15	p<0.001<br>r= 0.68	p<0.001<br>r= 0.83	p= 0.234<br>r= 0.20	p= 0.002<br>r= 0.50	p= 0.001<br>r= 0.52	p= 0.662<br>r= -0.075	p= 0.261<br>r= 0.19	p= 0.004<br>r= 0.46	p= 0.269<br>r= -0.19	p= 0.001<br>r= 0.54	p= 0.916<br>r= -0.018	p= 0.01<br>r= 0.42	p= 0.014<br>r= 0.41	p= 0.007<br>r= 0.44	p= 0.335<br>r= -0.17	p= 0.129<br>r= 0.26	pfkpa	p= 0.898<br>r= -0.022	p= 0.301<br>r= -0.18
p= 0.142<br>r= 0.25	p= 0.165<br>r= -0.24	p= 0.269<br>r= -0.19	p= 0.943<br>r= 0.012	p= 0.516<br>r= 0.11	p= 0.02<br>r= -0.39	p= 0.761<br>r= 0.052	p= 0.859<br>r= -0.031	p= 0.037<br>r= -0.35	p<0.001<br>r= 0.63	p= 0.217<br>r= -0.21	p= 0.292<br>r= 0.18	p= 0.724<br>r= -0.061	p= 0.045<br>r= 0.34	p= 0.281<br>r= 0.18	p= 0.193<br>r= 0.22	p= 0.971<br>= -0.0062	p= 0.381<br>r= -0.15	sema3d	p= 0.677<br>r= 0.072
p= 0.012<br>r= 0.41	p= 0.205<br>r= 0.22	p= 0.498<br>r= 0.12	p= 0.441<br>r= 0.13	p= 0.024<br>r= 0.38	p= 0.091<br>r= -0.29	p= 0.001<br>r= 0.52	p= 0.029<br>r= 0.36	p= 0.6<br>r= -0.09	p= 0.081<br>r= 0.29	p= 0.998<br>r= -0.0004	p= 0.074<br>r= 0.30	p= 0.629<br>r= -0.083	p= 0.001<br>r= 0.53	p= 0.91<br>r= -0.02	p= 0.339<br>r= 0.16	p= 0.751<br>r= 0.055	p= 0.483<br>r= -0.12	p= 0.165<br>r= 0.24	wnt5b

Supplementary Figure 1:

Pairwise gene expression correlations among the 20 candidate genes in the dorsal fin (upper diagonal) and the anal fin (lower diagonal). Pearson correlation coefficients (r-values) and p-values are shown. Blue font highlights significantly ($p < 0.01$) positive correlations; red font highlights significantly ($p < 0.01$) negative correlations.

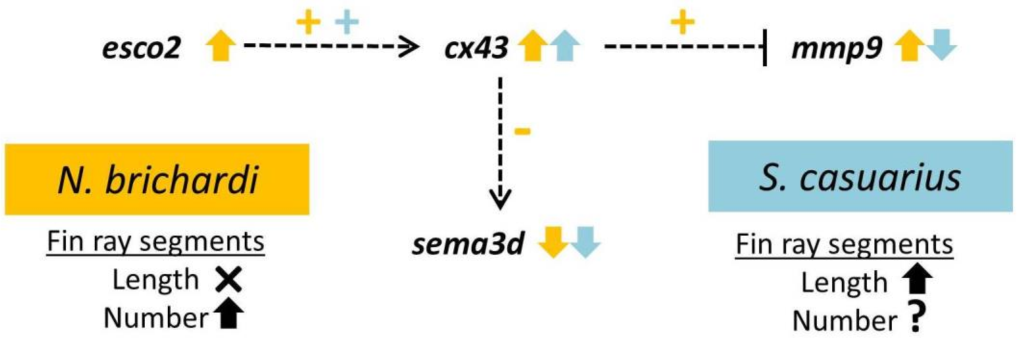

Supplementary Figure 2:

Gene expression patterns in elongated fins of the East African cichlid species *Neolamprologus brichardi* and *Steatocranus casuarius*, mapped onto a regulatory axis involved in fin ray segment formation in the zebrafish. The black, dotted lines reflect the regulatory interactions inferred in zebrafish. Above these lines, colored symbols indicate statistically significant positive (+) and negative (-) expression correlations between genes in the cichlid species (yellow, *N. brichardi*; blue, *S. casuarius*). Statistically significant gene expression differences between elongated and short fin regions in the cichlid species are indicated by the upward and downward arrows next to the genes' names, signifying significantly higher or lower expression levels, respectively, in the elongated fin region. Anatomically, fin elongation in *N. brichardi* is achieved by increasing the number, but not the length, of fin ray segments, whereas in *S. casuarius*, elongated fin rays have longer segments than short fin rays.

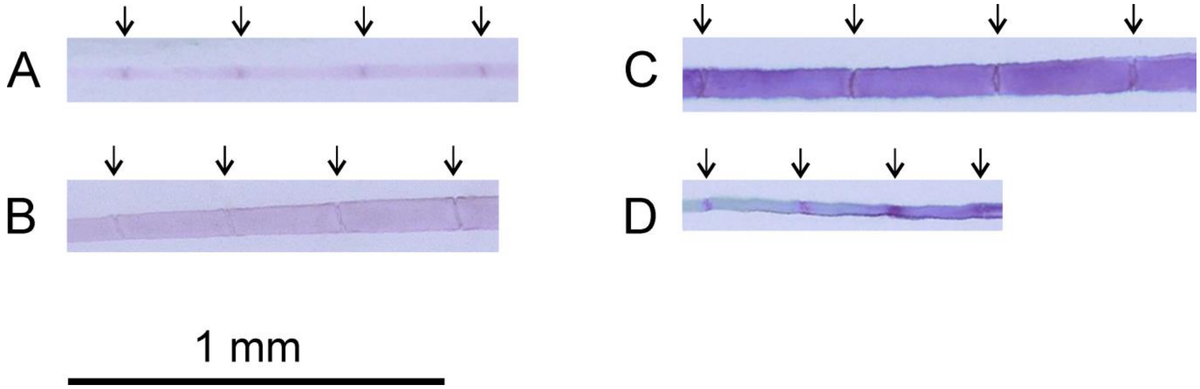

Supplementary Figure 3:

Examples of fin ray segments in the elongated and short regions of the dorsal fins of *Neolamprologus brichardi* and *Steatocranus casuarius*. (**A**) elongated fin ray of N. brichardi; (**B**) short fin ray of *N. brichardi*; (**C**) elongated fin ray of *S. casuarius*; (D) short fin ray of *S. casuarius*. Arrows mark the segment borders.
