# Supplementary material for: Gene expression profiling suggests differences in molecular mechanisms of fin elongation between cichlid species
Source: Sci Rep. 2019 Jun 21;9:9052. doi: 10.1038/s41598-019-45599-w (PMC6588699; doi:10.1038/s41598-019-45599-w)
Supplement: Supplementary file 2 — Supplementary Table 1 [file 41598_2019_45599_MOESM2_ESM.pdf]

Gene expression profiling suggests differences in molecular mechanisms of fin elongation between cichlid species

Ehsan Pashay Ahi[1,2],
Email: ehsanpashayahi@gmail.com

Florian Richter[1]
Email : florianrichter1@outlook.com

Laurène Alicia Lecaudey[1],
Email: laurenelecaudey@gmail.com

Kristina M. Sefc[1],
Email: kristina.sefc@uni-graz.at

1. Institute of Biology, University of Graz, Universitätsplatz 2, A-8010 Graz, Austria

2. Department of Comparative Physiology, Uppsala University, Norbyvägen 18A, SE-75 236 Uppsala, Sweden

Corresponding Author: Ehsan Pashay Ahi,
Email: ehsanpashayahi@gmail.com

Supplementary Table 1:

Ray segment length differences between fin rays. Upper diagonal: fin ray comparisons in the anal fin; lower diagonal: fin ray comparisons in the dorsal fin. Significant length differences are highlighted in bold.

		**Anal fin**			
		Long ray 1	Long ray 2	Short ray 1	Short ray 2
**Dorsal fin**	Long ray 1	-	**t = -3.18, p = 0.002**	**t = -2.58, p = 0.011**	**t = -3.07, p = 0.002**
	Long ray 2	t = -0.01, p = 0.99	-	t = 0.60, p = 0.55	t = 0.11, p = 0.91
	Short ray 1	**t = 8.15, p < 0.001**	**t = 8.16, p <0.001**	-	t = -0.49, p = 0.63
	Short ray 2	**t = 10.09, p < 0.001**	**t = 10.10, p <0.001**	t = -1.94, p = 0.054	-

Long ray 1: longest ray of each fin; long ray 2: 2nd-longest ray; short ray 1: 3rd-shortest ray; short ray 2: 2nd-shortest ray (the very shortest ray was avoided because it was only rudimentarily developed in some fins).
